# Adjunctive Punctal Re-Dilation for Early Postoperative Cicatrization after One-Snip Punctoplasty

**DOI:** 10.14744/bej.2021.59251

**Published:** 2021-12-17

**Authors:** Fatma Corak Eroglu, Emine Sen, Burcu Kazancı

**Affiliations:** Department of Ophthalmology, University of Health Sciences, Ulucanlar Eye Training and Research Hospital, Ankara, Turkey

**Keywords:** Epiphora, one-snip punctoplasty, punctal re-dilatation, punctal stenosis, re-stenosis

## Abstract

**Objectives:**

This was an assessment of one-snip punctoplasty outcomes in patients for whom adjunctive punctal re-dilatation was performed in-office for early postoperative cicatricial changes.

**Methods:**

A retrospective analysis was conducted of patients who underwent one-snip punctoplasty between March 2019 and February 2020 due to acquired punctal stenosis. Patients were followed up on the first, third, and seventh postoperative day, then weekly for the remainder of the first month, every 2 weeks over the next month, and then monthly. Punctal re-dilatation was performed if patients showed early clinical signs of re-stenosis. Demographic details, the number and timing of re-dilatation procedures, the timing of re-stenosis, and anatomical and functional success rates were analyzed.

**Results:**

The medical records of 148 eyes of 86 patients were evaluated. A re-dilation procedure was performed in a total of 57 (38.5%) puncta showing signs of early cicatrization. The first punctal re-dilatation was performed at a mean of 17.2±11.3 days (range: 3–57 days). Re-stenosis was observed in 25 puncta (16.9%) at a mean of 5.6±3.1 weeks (range: 2–16 weeks). The anatomical success rate was 83.1% and the functional success rate was 79.1%. There were no significant differences in the anatomical and functional success rates between the patients who did and did not need adjunctive re-dilatation.

**Conclusion:**

In-office punctal re-dilatation may improve functional and anatomical success rates after one-snip punctoplasty by preventing recurrent punctal cicatrization.

## Introduction

Acquired external punctal stenosis (AEPS) is a common lacrimal disorder that causes epiphora because of the blockage of the lacrimal pathway (1). It has been associated with a variety of causes, including conjunctivitis, chemical burns, systemic chemotherapy, ocular medications, chronic blepharitis, and advanced age (1-4). Despite this long list, the most of the cases remain idiopathic. The basic principles in the treatment of AEPS include creating an adequate opening without re-stenosis, maintaining the punctal position, and preserving the lacrimal pump function (5).

Although many procedures have been described to overcome this condition, including the punctal dilatation, punctoplasty procedures, punctum pucker procedure, posterior punctectomy with intraoperative mitomycin C, punctal punching, punctal plug, silicone stenting, and balloon punctoplasty, there is continued debate over what constitutes the most efficacious way to perform the procedure (1, 5-17).

Punctoplasty was introduced in 1853 by Bowman (6) and in 1962 by Jones (7) re-popularized one-snip punctoplasty, and he suggested periodic punctal dilatation to prevent wound re-approximation and fibrosis-related failure if the punctum is showing to re-stenosis findings. Despite this historical information in the literature (1, 6-8), there is a lack of large-scale study involving the results of one-snip punctoplasty with adjunctive punctal dilatation for early post-operative cicatricial changes. The one- snip punctoplasty, which a single vertical snip down the ampulla, has been reported to have a failure because of recurrence secondary to wound re-approximation (6-11, 18). Jones (7) suggested that if the punctum tenting to re-stenosis after one snip punctoplasty procedure, successful results can be achieved with periodic punctal dilation. However, there is a lack of study published on failure and success rates of one-snip punctoplasty assisted by punctal re-dilatation procedure. In 1986, Dolin and Hecht (11) introduced the punctum pucker procedure to prevent re-approximation of cut raw ends and reported 100% success in their eight cases for 6months follow-up. They reported that puckering the punctum during post-operative 3–5 days can prevent re-epithelization of the cut ends. However, they did not emphasize whether there was a sign of re-approximation or its timing.

The aim of the study was to present the outcomes of one-snip punctoplasty with adjunctive in-office dilatation for early post-operative cicatricial change. We also investigated the timing of the sign of cicatrization and the factors that may be associated with re-stenosis. To our knowledge, this is the largest study presenting outcomes of one-snip punctoplasty assisted with punctal re-dilatation and investigated associated factors for early cicatrization and re-stenosis.

## Methods

Medical records of 169 eyes of 98 consecutive patients undergoing one-snip punctoplasty by a single oculoplastic surgeon (FCE) in a tertiary center between March 2019 and February 2020 were analyzed retrospectively. The study was approved by the local Institutional Ethics Committee, and written informed consent was taken from every participant. Declaration of Helsinki was followed throughout the study.

The patients with associated canalicular or nasolacrimal duct obstruction, congenital punctum disorders, eyelid malposition, systemic or topical chemotherapy, and previous interventions to the lacrimal system or eyelid did not include the study. Furthermore, case with combined procedures, such as lid position correction or canalicular intervention, were excluded to avoid confounding factors. We only included case with post-operative follow-up longer than 6 months.

The patients underwent an ophthalmic examination to reveal underlying causes and were questioned about the onset of their symptoms. The patients with AEPS after treating the blepharitis were undergone one-snip punctoplasty. Medical history and ocular and systemic medication records were obtained. PS was defined on slit-lamp examination and a diagnostic probing. The one-snip procedure has been considered when the following criteria were met: Narrowing puncta, the patient with symptomatic epiphora, patent lacrimal syringing, and no other causes of epiphora.

One-snip punctoplasty was done by a single oculoplastic surgeon (FCE) in an operating room with the aid of a microscope under local anesthesia. Lacrimal probing and irrigation through the normal punctum were performed to exclude concomitant canalicular, common canalicular, or nasolacrimal obstructions. After installation of topical anesthetic drop (Proparacaine HCl 0.5%), 1 ml local anesthetic (Lidocaine 2% with adrenaline 1:10,000) was injected around the punctum and canaliculus. After dilatation of the punctum, one vertical incision was made through the posterior wall of the punctum and vertical canaliculus. To prevent induced fibrosis, the conjunctiva in the surgical site was preserved, and hemostasis was achieved with pressure. Postoperatively, all patients were treated with a topical antibiotic (Levofloxacin 0.5%, 4×1 a day) and steroid (Fluorometholone acetate 0.1%, 4×1, a day) for 10 days.

Follow-up examination was performed postoperatively on the 1^st^, 3^rd^, and 7^th^ day, and then weekly during the 1^st^ month, every 2 weeks over the next month, and then monthly. At each visit, punctum was examined in terms of whether re-stenosis to started. A punctal re-dilatation was performed in the examination chair if patients showed early clinical signs of fibrosis and cicatrization. It was performed under topical anesthetic drop using a 26-gauge lacrimal dilator and advanced to the vertical canaliculus to release the cut ends of the punctum.

The collection of data has consisted of demographic detail, duration of symptoms, underlying causes, laterality, first observed time of cicatrization, the number and timing of re-dilatation, and timing of re-stenosis. Anatomical success was defined as visible patent punctum and functional success as absence of epiphora at the last post-procedure visit. The functional and anatomical success, timing of early cicatrization, and timing and rates of re-stenosis were analyzed. We also compared the patients who needed and did not need re-dilatation in terms of surgical success and recurrence rates.

### Statistical Analysis

The SPSS software version 22.0, IBM, Chicago, IL, USA, was used for statistical analysis. Descriptive statistics, including the mean, standard deviation and range were calculated for different variables. The Chi-square and Fisher’s exact tests were used for statistical analysis. A p<0.05 was considered statistically significant.

## Results

One hundred-sixty-nine puncta of 169 eyes of 98 patients satisfied the inclusion criteria. However, 21 eyes of 12 patients were excluded from the study due to a lack of follow-up. The medical records of 148 eyes of 86 patients were evaluated. The demographic detail of the patients summarized in Table 1. The period between the onset of symptoms and the surgery was 5.7 + 7.9 months (1–15 months), and the follow-up length ranged from 9 to 22 months with a mean of 13.6 + 3.1 months. A total of 32 eyes (21.7%) had a history of topical ophthalmic medication before onset of symptoms. Chronic blepharitis was diagnosed in 62 (41.9%) eyes, with all of them had bilateral involvement of PS. Because of the retrospective design of the current study, the number of the patients could not give an accurate medical history or previous medication, and thus 36.4% of the eyes remained as unknown etiology.

**Table 1.** Patients’ demographics

**Primary punctoplasty procedures identified (n)**	**148**
Patients (n)	86
Male/Female (patients), n (%)	30 (34.9)/56 (65.1)
Mean age at surgery (years)^*^	56±8.6
Underlying or associated causes	
Blepharitis (eyes), n (%)	62 (41.9)
Topical medication (eyes), n (%)	
Latanoprost	4 (2.7)
Timolol	2 (1.4 )
Fixed combination of brimonidine and timolol	4 (2.7)
Moxifloxacin drop	8 (5.4)
Prednisolone acetate drop	5 (3.4)
Unknown topical medication	9 (6.1)
Undetectable etiology (punctum), n (%)	54 (36.4)
Mean symptom duration (months)^*^	5.7±7.9
Patients undergoing bilateral surgery, n (%)	62 (72.1)
Mean follow up (months)^*^	13.6±3.1
Range of follow-up (months)	9–22
Side of surgery (right/left), n (%)	76 (51.4)/72 (48.6)

*Mean values with statandard deviation (±).

Table 2 included the outcomes of the surgery. After surgery, a total of 57 punctum (38.5%) showed signs of early cicatrization and underwent re-dilatation at least once. The number of mean dilatations was 1.73 + 0.6 times (range 1–3). The mean time of the first dilatation was 17.2 + 11.3 days (3–57 days).

**Table 2.** Clinical outcomes of one-snip punctoplasty procedure

Need for re-dilatation (punctum), % (n)	38.5% (57)
Number of dilatation (mean±SD), (range)	1.73±0.6 (1–3)
First dilatation timing^*^ (days), mean±SD, (range)	17.2±11.3 (3–57)
Number and rate of re-stenosis (punctum), % (n)	16.9% (25)
Timing of re-stenosis (weeks), mean±SD, (range)	5.6±3.1 (2–16)
Anatomical success, %, (n)	83.1% (123)
Functional success, % (n)	79% (117)

^*^First dilatation timing: Time when early cicatrization was first observed.

Overall anatomical and functional success rates of one-snip punctoplasty were detected as 83.1% (123) and 79% (117), respectively. The re-stenosis was observed in 25 puncta (16.9%) at an average of 5.6 + 3.1 weeks (2–16 weeks). When the compare the patients, who needed adjunctive re-dilatation and those who did not, there was no significant difference in re-stenosis, anatomical and functional success rates (p<0.05 for each values) (Table 3).

**Table 3.** Comparison of surgical success and recurrence rates of the patients with and without adjunctive punctal re-dilatation

**The patients in need**	**Adjunctive re-dilatation (n=57 puncta)**	**The patients without the need in adjunctive re-dilatation (n=91 puncta)**	p^*^
	**n**	**%**	**n**	**%**	
Anatomical success	47/57	82.5	76/91	83.5	0.52
Functional Success	45/57	78.9	72/91	79.1	0.49
Punctal re-stenosis	10/57	17.5	15/91	16.5	0.5

*Fisher exact test.

## Discussion

In this study, we intent to present in the largest series the results of one-snip punctoplasty with adjunctive punctal re-dilatation for early post-operative cicatricial changes and to investigate re-dilatation need and the timing of re-stenosis. Although almost 40% of the eyes showed findings of early re-approximation, the re-stenosis rate remained at 16.9% at an average 13.6 months follow-up. We achieved 83.1% anatomical and 79.1% functional success rates overall.

There is no study in the literature reporting when the raw cut ends of the punctum show to re-approximation after any punctoplasty surgery. In the present study, the re-approximation of raw cut ends was observed between the range of the 3^rd^ day (second visit) and the 57^th^ day (eighth visit). The interval between the time of re- approximation of cut ends (mean: 17.2 days) and punctal re-stenosis (mean: 5.6 months) was quite wide. We also found similar re-stenosis rates in the patients both undergone adjunctive re-dilatation and those who did not. The re-approximation could be explained by re-epithelization, which means the failure of one –snip procedure; however, the re-stenosis or re-approximation observed later may be regardless of the failure of the procedure. However, this distinction is difficult to make.

It is known that punctal re-dilatation is an additional procedure that can be combined with any punctoplasty procedure when patients showed signs of cicatrization at their postoperative follow (7, 20). Fraser et al. (20) performed three-snip punctoplasty and adjunctive punctal re-dilatation for all patients in their study. Despite their anatomical and functional success rates were higher, compared to our study, their mean follow-up (mean: 4.4 months) was short, and the population of the study group (28 punctum of 22 patients) was smaller than our study. Different from their work, we performed adjunctive re-dilatation only in patients with early re-approximation. We found similar anatomical and functional success rates in patients with and without signs of re-approximation of cut ends. It means functional and anatomical success rates can be improved by in-office re-dilation in patients with early signs of punctal cicatrization after the one-snip procedure.

Due to the advantages and disadvantages of all these methods described, it is difficult to establish a standard procedure for the treatment of AEPS (6-11, 18, 19) Among various snip procedures described, the three-snip punctoplasty is the most popular and has been documented with high success rates (8-10, 18, 19). However, it is believed to cause damage to both vertical and horizontal canaliculus, and consequently lacrimal pump function (1, 8, 9). The anatomic success varied from 31 to 94%, and the functional success varies from 18 to 81% between studies (8-10, 19). Rectangular punctoplasty, which is claimed to preserve the normal anatomy and physiology of the canalicular system, was introduced in 2009, and success rates varied from 74.7% to 93.3% (1, 18, 21). The Mini-Monoka insertion has been advocated to prevent re-approximation of cut ends and preserve punctum anatomy. Despite the high functional success rates reported (from 82 to 88%), its main drawback is the remarkable premature stent loss, increase operating time, patient discomfort, infectious rates, and cost (1, 16).

When we investigate of 25 anatomical failure puncta, 10 eyes had chronic blepharitis, two eyes had a history of topical anti-glaucoma medication, two patients (four eyes) were smoker, and in nine eyes did not be found any etiological factors. We also found six eyes had symptomatic epiphora despite patent puncta and lacrimal pathway. Three eyes of two patients had recurrence chronic blepharitis due to lack of lid hygiene and inadequate treatment of patients. One of them (two eyes) was a heavy smoker for 30 years, and a definite causative factor could not be indicated in one eye.

In the current study, with the female predominance, the mean age was 56.9 with consistent with the literature. Involutional changes can reason the dense fibrous structure of the punctum to become less flexible and surrounding orbicularis fibers to become atonic, resulting in PS (1, 3, 4). Although still controversial, the female gender has been proposed as a risk factor for developing AEPS on the basis of hormonal changes (1, 3). We primarily focused on the results of one-snip punctoplasty in this study, and the cases with associated lid malposition or concomitant canalicular stenosis were excluded. Due to the retrospective nature of our study, the exact and accurate medical history of some patients could not be gained, so significant number of the stenotic punctum (36.4%) remained idiopathic. In the rest of them, chronic blepharitis was the most common detectable underlying cause in our series (41.9%). In various studies, association of AEPS and chronic blepharitis have been reported from 45 to 64.3% based on inflammatory and cicatricial change over the external punctum (1, 3-5, 22). In our series, a total of 32 eyes (21.7%) had a history of topical medications before the onset of symptoms. It has been reported that AEPS may be caused by the toxic effect of ophthalmic medication, publications are reporting up to 75% (1, 4, 23, 24).

To our knowledge, this is the largest series to reporting the long-term results of one-snip punctoplasty assisted by punctal re-dilatation for early post-procedure cicatrization. We are also able to demonstrate for the first time in the literature the time of early cicatrization of the cut ends and the timing of re-stenosis. Considering that early re-approximation of cut ends has developed within an average of 17.2 days, re-stenosis can be reduced by closely monitoring the patients for the first 3 weeks after surgery and re-dilatation when necessary.

The current study is limited by being retrospective analyses of a single technique. Although it cannot be said to be more effective, we propose that adjunctive re-dilatation is a reasonable means for improving post-operative success in one-snip punctoplasty procedure. Additional prospective assessments are needed to evaluate the efficacy of this adjunctive procedure after one-snip punctoplasty. Another limitation of the study was that this procedure required frequent visits. The patients with AEPS have been suffered from epiphora for a long time, and they visited different times many ophthalmologists. Hence, when we explained the risk of re-stenosis after the procedure, frequent follow-ups did not negatively affect the patient’s compliance in the most cases.

## Conclusion

In the study in which we presented the long-term results of one-snip punctoplasty, the mean time of re-stenosis was found to be 17.2 days. Despite the general opinion that one-snip punctoplasty has high re-stenosis rates, we emphasize that acceptable success rates can be achieved by close monitoring patients and performing adjunctive re-dilation when needed. It may be introduced as an initial treatment for the primary treatment of AEPS before applying to more complicated and expensive procedures.

### Disclosures

**Ethics Committee Approval:** Anakara Training and Research Hospital Ethics Committee, protocol number: E-Kurul-E-20, Date: 17/09/2020.

**Peer-review:** Externally peer-reviewed.

**Conflict of Interest:** None declared.

**Authorship Contributions:** Involved in design and conduct of the study (FCE); preparation and review of the study (FCE, ES); data collection (FCE, ES, BK); and statistical analysis (FCE).
